# Diversified Microbiota of Meconium Is Affected by Maternal Diabetes Status

**DOI:** 10.1371/journal.pone.0078257

**Published:** 2013-11-06

**Authors:** Jianzhong Hu, Yoko Nomura, Ali Bashir, Heriberto Fernandez-Hernandez, Steven Itzkowitz, Zhiheng Pei, Joanne Stone, Holly Loudon, Inga Peter

**Affiliations:** 1 Department of Genetics and Genomic Sciences, Icahn School of Medicine at Mount Sinai, New York, New York, United States of America; 2 Department of Psychiatry, Icahn School of Medicine at Mount Sinai, New York, New York, United States of America; 3 Department of Psychology, Queens College, CUNY, Flushing, New York, United States of America; 4 Division of Gastroenterology, Department of Medicine, Icahn School of Medicine at Mount Sinai, New York, New York, United States of America; 5 Department of Veterans Affairs New York Harbor Healthcare System, New York, New York, United States of America; 6 Department of Pathology, NYU Langone Medical Center, New York University School of Medicine, New York, New York, United States of America; 7 Department of Obstetrics, Gynecology, and Reproductive Sciences, Icahn School of Medicine at Mount Sinai, New York, New York, United States of America; The University of Hong Kong, Hong Kong

## Abstract

**Objectives:**

This study was aimed to assess the diversity of the meconium microbiome and determine if the bacterial community is affected by maternal diabetes status.

**Methods:**

The first intestinal discharge (meconium) was collected from 23 newborns stratified by maternal diabetes status: 4 mothers had pre-gestational type 2 diabetes mellitus (DM) including one mother with dizygotic twins, 5 developed gestational diabetes mellitus (GDM) and 13 had no diabetes. The meconium microbiome was profiled using multi-barcode 16S rRNA sequencing followed by taxonomic assignment and diversity analysis.

**Results:**

All meconium samples were not sterile and contained diversified microbiota. Compared with adult feces, the meconium showed a lower species diversity, higher sample-to-sample variation, and enrichment of *Proteobacteria* and reduction of *Bacteroidetes*. Among the meconium samples, the taxonomy analyses suggested that the overall bacterial content significantly differed by maternal diabetes status, with the microbiome of the DM group showing higher alpha-diversity than that of no-diabetes or GDM groups. No global difference was found between babies delivered vaginally versus via Cesarean-section. Regression analysis showed that the most robust predictor for the meconium microbiota composition was the maternal diabetes status that preceded pregnancy. Specifically, *Bacteroidetes* (phyla) and *Parabacteriodes* (genus) were enriched in the meconium in the DM group compared to the no-diabetes group.

**Conclusions:**

Our study provides evidence that meconium contains diversified microbiota and is not affected by the mode of delivery. It also suggests that the meconium microbiome of infants born to mothers with DM is enriched for the same bacterial taxa as those reported in the fecal microbiome of adult DM patients.

## Introduction

Prenatal diabetes, which includes both pre-gestational Type 1 and Type 2 diabetes, and gestational diabetes that develops during pregnancy have been associated with an increased risk of obstetric and neonatal complications [1]–[3]. Both pre-gestational and gestational diabetes have also been associated with major birth defects [4] and congenital anomalies of the offspring [5], [6]. Moreover, gestational diabetes has been linked to the risk of childhood obesity [7], [8], which has both immediate and long-term implications on human health.

Human microbiome studies have demonstrated dynamic changes in bacterial composition in the gut during pregnancy and childhood development [9]–[11]. Moreover, the presence of pathogenic species, or absence of beneficial species, in early childhood has been suggested to play a key role in the initiation of preterm birth [12], development of asthma or eczema [13], [14], allergy [15], autism [16] or other immunological deficiency [17], [18].

Historically, the fetus, as well as the intrauterine environment, has been considered sterile, with the initial microbial exposure taking place at birth vaginally or via C-section through contacting maternal vaginal or skin microbiota, respectively [9], [11], [19], [20]. However, accumulating evidence suggests the presence of different microbes in amniotic fluid [21], [22], umbilical cord blood [23], meconium [24], [25], and placental [26] and fetal membranes [27]. Studies in mice have demonstrated the transmission of labeled bacterial strains from a mother to fetus during pregnancy [25]. Taken together, these results suggest that mother-to-baby efflux of commensal microbes may occur prior to birth.

However, despite the growing recognition that commensal microbes may contribute fundamentally to infant and childhood development and immunity [14], [15], [17], [18], [28], [29], only a few studies have determined the microbial composition of the first intestinal discharge, or meconium, in premature [30]–[32] and in term neonates [24], [25] and linked its bacterial content to maternal eczema and infant mucus congestion during the first year of life [24]. Therefore, the main objectives of this study were to further characterize the composition of the meconium and assess whether maternal diabetes status, prior to or during pregnancy, affects bacterial composition of the newborn's first stool.

## Methods

### Subjects

This study was approved by the Mount Sinai Institutional Review Board. Pregnant women before their second trimester were recruited during their regular prenatal visits at a prenatal obstetrics and gynecological (OB/GYN) clinic at Mount Sinai Medical Center and provided a written informed consent for themselves and their prospective infants. The exclusion criteria included: 1) any antibiotic treatment during pregnancy; women who eventually underwent C-section and received an immediate dose of Kefzol (cefazolin) <30 mins prior to C-section as a standard of care were retained in the study, or 2) obstetric risks, such as HIV positivity, significant congenital anomalies, neurological dysfunction, fetal chromosomal anomalies, or inborn errors in metabolism. Clinical characteristics of the infant included sex, birth weight (BW), birth length, time of sampling (hours after birth), neonatal complications, gestational age, and delivery method. Clinical variables of the mother included age, body mass index (BMI) at 1st and 3rd trimester, glucose level (1 hour glucose challenge test, or GCT, completed at 24–28 weeks), medications during pregnancy, maternal smoking, and diabetes status. A subclinical group included 4 no-diabetes mothers with glucose levels higher than the cut-off point of 130 ng/dL, who did not meet the criteria for gestational diabetes at the diagnostic 3-hour glucose tolerance test (GTT). Such women are known to carry additional obstetric risk, such as fetal macrosomia and other morbidities [33], [34]. Seven adult fecal samples used for the comparison purposes in the present study were collected for an unrelated study from healthy individuals with no diabetes and no antibiotic treatments for at least 6 months, who consented for their samples to be used for other research.

### Sample collection

The neonate meconium from 23 enrolled infants was passed ranging between 2 hours and 48 hours after birth (**Table 1**). The meconium was transferred from a diaper to sterile 15 ml Falcon tubes using a sterile tongue depressor by the research staff and stored at −80°C until processing. During the sample collection and processing, the mother did not handle the meconium in order to avoid possible contaminations.

**Table 1:** Clinical information of the neonates and mothers.

	Infant								Mother				
IDs	Sex	BW^1^ (gram)	Length (cm)	Complication & Treatment	TOS^2^ (hrs)	GADAYs^3^ (days)	Delivery Status	Type of C-section	BMI1^4^	BMI2^5^	Maternal Age	Glucose Level	Diabetic Status
BM62	F	1645	52	NICU (dextrose, fat emulsion, multivitamins)	24	259	cesarean	Emergency, Induced labor	42.45	40.78	29	106	HC^6^
BM110	F	3450	51		6	275	cesarean	Emergency, Arrest of dilation	30.11	34.96	24	93	HC
BM172	F	3690	52.5		43	287	vaginal		23.17	27.56	34	96	HC
BM124	M	4060	53		2	291	cesarean	Emergency, Arrest of dilation	27.04	33.12	23	120	HC
BM158	F	3505	51		n/a	283	vaginal		19.38	23.58	21	75	HC
BM38	F	3370	48		20	272	vaginal		20.67	21.77	30	117	HC
BM188	F	3410	48.5		27	285	vaginal		19.00	26.30	17	81	HC
BM50	F	2500	48		25	279	vaginal		30.00	37.19	24	90	HC
BM177	F	3246	51		9	280	vaginal		26.94	32.90	20	139	HC
BM3	M	3485	53		5	274	vaginal		25.70	30.30	26	168	Subclinical
BM180	F	3040	49		12	255	cesarean	Elective, Induced labor	30.90	34.40	39	141	Subclinical
BM52	M	2660	49		15	251	vaginal		26.08	28.96	23	164	Subclinical
BM276	F	2875	49.5		8	291	vaginal		n/a	44.07	39	165	Subclinical
BM68	M	2630	49	NICU (bacitracin ointment, nystatin, phenobarbital, rantidine, simethicone, dextrose, ferrous sulfate, multivitamins)	48	250	cesarean	Emergency, Arrest of descent	22.90	23.63	27	285	GDM^7^
BM126	F	3410	52	Jaundice	4	280	vaginal		35.02	40.96	28	151	GDM
BM9	M	3240	56		4	266	vaginal		21.17	34.92	37	92	GDM
BM256	F	3800	51		9	282	vaginal		n/a	31.82	31	131	GDM
BM257	F	4000	52		16	266	cesarean	Emergency, Arrest of dilation	n/a	39.45	42	182	GDM
BM25	F	2770	48		2	291	cesarean	Elective, Induced labor	27.04	33.12	23	120	DM^8^
BM181	M	3120	50.5		45	249	vaginal						DM
BM155A	M	3125	50		24	233	cesarean	Emergency, Fetus stress	n/a	27.5	41	n/a	DM
BM155B	M	1750	40.5	NICU (palivizumab, lidocaine-prilocaine cream, ferrous sulfate, dextrose, artificial tears, multivitamins)	24	233	cesarean	Emergency, Fetus stress	n/a	27.5	41	n/a	DM
BM140	M	2940	50	NICU (lidocaine-prilocaine cream, lidocaine injection, artificial tears)	40	270	cesarean	Emergency, Induced labor	23.84	25.95	23	n/a	DM

1 BW = body weight;

2 TOS = Time of sample collection;

3 GADAYs = gestational age in days;

4 BMI1 = body mass index(BMI) in 1^st^ trimester;

5 BMI2 = body mass index(BMI) in 3^rd^ trimester;

6 HC = Healthy control;

7 GDM = gestational diabetes;

8 DM = Type 2 diabetes.

### Fecal DNA extraction and 16S ribosomal RNA (rRNA) amplification

Total meconium DNA was extracted using Qiagen stool kit (Qiagen, CA). Subsequent amplification of bacterial 16S rRNA v3–v4 region using barcoded PCR primers (**Table S1 in File S1**) was performed as previously described [35]. A 16-mer error-correcting Golay barcode was added to the reverse primer. A composite bar-coded sample for sequencing was created by combining equimolar amounts of amplicons from the 23 individual samples.

### 16S rRNA sequencing, taxonomy assignment and diversity computation

The pooled 16S rRNA PCR amplicons were sequenced on the Pacbio RS system. Two 45-minute movies were collected on each SMRT cell. Analysis was performed only on circular consensus (CCS) reads (CCS reads are generated when ≥3 full pass subreads are present). Final CCS reads were filtered by the sequencing quality score and read length before being exported to FASTA format. To test the sequencing accuracy and reproducibility, three healthy adult stool samples were processed twice and sequenced on two separated SMRT cells. The *E.coli* strain BL21 was sequenced to test the classification accuracy. Filtered high quality CCS reads were further processed by QIIME 1.5.0 [36]. The alpha-diversity was calculated using the Shannon Index as metric and represented the mean species diversity within each sample. The beta diversity was estimated using both weighted and unweighted UniFrac distance matrices and represented the measurement of compositional dissimilarity among samples.

### Data Analysis

Wilcoxon-Mann-Whitney test was performed at various OTU (operational taxa unit) levels to compare between the adult feces and meconium with regard to and regardless of maternal diabetes status. *P*-values were adjusted for false-discovery rate (FDR) using the Benjamini and Hochberg method [37]. Non-parametric multiple dimensional scaling (nMDS) and Principal Coordinate Analysis (PCoA) were used to visualize the dissimilarities of the overall microbiome. The PerMANOVA test [38], [39], with the maximum number of permutations = 999, was performed using the [Adonis] function of the *R* package *vegan 2.0–5* [40] to compare the overall microbiome differences between the meconium microbiota by maternal diabetes status or type of delivery. We also performed the univariate regression analysis using the [lm] function in *R*.

The dependent variables included the unweighted principle components (PC): PC1, PC2 and PC3, representing the dominant taxa *Bacteroidetes*, *Firmicutes* and *Proteobacteria* at the phyla level and the unweighted UniFrac distance matrices representing the overall microbiome profile. The predictor included newborn sex, time of sampling, gestational age, delivery method, maternal BMI at 1^st^ trimester and maternal diabetes status.

## Results

### Clinical information

Twenty-three newborns were enrolled in this study (**Table 1**). Five of the babies were born to mothers with established DM, 5 to mothers with GDM, and 13 to mothers with subclinical diabetes (n = 4) or no diabetes (n = 9). One of the mothers with DM had dizygotic twins. None of the study participants reported being smokers. Ten babies were delivered by C-section with the type of C-sections (emergency or elective), as well as additional participant characteristics, listed in **Table 1**. No significant difference was observed for TOS, BW and length among DM, GDM, and no-diabetes groups (p-value >0.1 by t-test). Missing data resulted from the fact that some mothers only consented to certain components of the study.

### Validation of the sequencing accuracy and reproducibility

In this study, on average, a single chip generated ∼55,000 post-filtered reads (mean mapped CCS read accuracy about 94%), of which ∼33,000 reads were longer than 2 kilobases (**Figure S1A in File S1**). **Figure S1B in File S1** demonstrates that the mean CCS read length was 443 base pairs (bp, ranging between 400 and 500 bp). We tested reproducibility of our 16S rRNA sequencing technique by comparing duplicate samples. Taxonomy assignment showed that, the correlation between the duplicates was within the range of 99.5% to 99.9% at any taxonomy level (**Figure S1C in File S1**). When we examined the bacterial composition of the control *E.coli* strain BL21, 99.5% of the CCS reads were correctly assigned at the phyla through family level and 97.1% at the genus level, suggesting a high accuracy and low misclassification rate (**Figure S1D in File S1**).

### Bacterial Composition of the Meconium and Comparison to the Adult and Infant Microbiome

**Figure 1:**
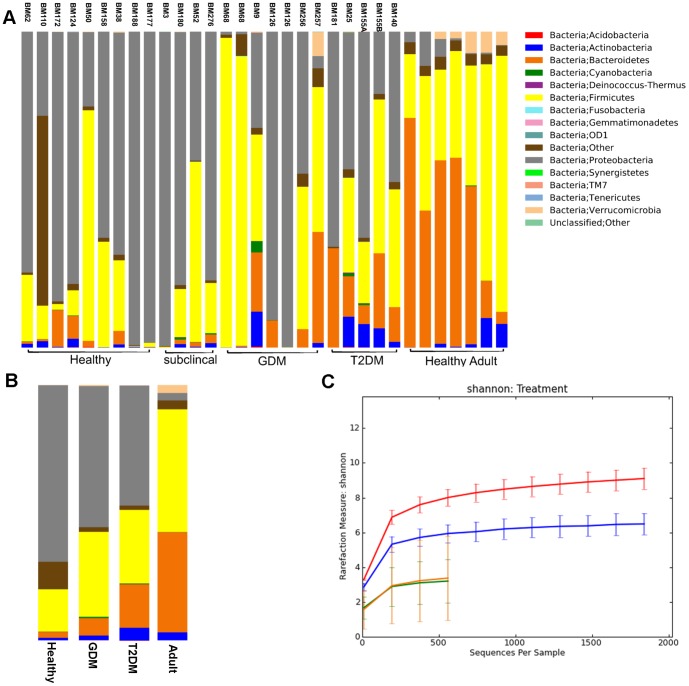
Comparison of the microbiota communities of neonate meconium to adult stool samples. A. Classification of the microbiota at the Phylum level. Samples BM62-BM140 are neonate meconium and 7 samples are from healthy adults. The total reads for each sample are normalized to 2000. Neonate meconium samples are grouped by maternal clinical status: Healthy, subclinical, GDM (gestational diabetes) and DM (type 2 diabetes). B. Average taxa abundances between the neonate meconium and adult stool samples. HC group combined healthy and subclinical samples. C. Alpha rare plot shows the average microbiota diversity of the neonate meconium grouped by maternal status (HC, GDM and DM) and adult stool samples.

**Figure 2:**
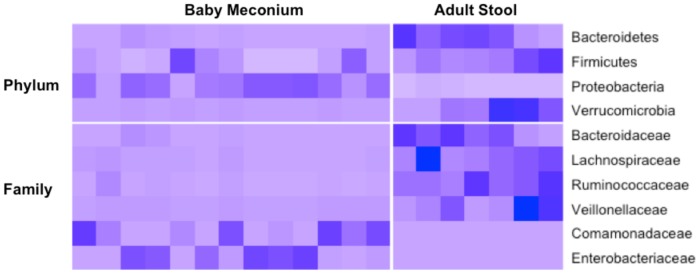
Comparison of the OTUs at the Phylum and Family level between healthy adult stool samples and the meconium samples from neonates born to mothers with no diabetes. The meconium samples included both HC and subclinical groups. Only OTUs shown significance (p values <0.05 by WMW-test and adjusted by BH method) between the two groups are presented.

After filtering by quality and length, we obtained, on average, 3,300 CCS reads per sample (range 1,464–6,641). Our results indicated that all 23 meconium samples were not sterile. At the phylum level, all four major phyla, *Actinobacteria*, *Bacteroidetes*, *Firmicutes* and *Proteobacteria*, were represented accounting for about 99% of the microbial content (**Figure 1A**). However, the average taxa abundance of the newborn meconium was very different from that of the adult stool samples (**Figure 1B**). Specifically, when compared with the adult feces (**Figure 2**), the meconium from the neonates born to no-diabetes (healthy and preclinical DM) mothers had a significantly higher proportion of *Proteobacteria* (71.0% in the meconium vs. 3.1% in the adult stool), and a lower proportion of *Bacteroidetes* (2.5% in the meconium vs. 42.8% in the adult stool), *Firmicutes* (20.0% in the meconium vs. 44.7% in the adult stool) and *Verrucomicrobia* (0.06% in the meconium vs. 3.2% in the adult stool) (FDR-adjusted *p*<0.05). At the family level, *Comamonadaceae* and *Enterobacteriaceae* were significantly enriched in the meconium samples compared to the adult stool (FDR-adjusted *p*<0.05). Further analyses showed a significant reduction in *Bacteroidaceae*, *Lachnospiraceae*, *Ruminococcaceae* and *Veillonellaceae* (FDR-adjusted *p*<0.05) in the meconium. The four most abundant genera in the meconium included *Comamonas*, *Escherichia/Shigella*, *Klebsiella* and *Proteus*. Similar to the no-diabetes group, the meconium from DM and GDM groups also showed enrichment of *Proteobacteria* and reduction of *Bacteroidetes* and *Firmicutes*, in comparison with the adult stool samples.

As the composition of the first stool is likely to change in response to environmental exposures, we compared the meconium microbiome content to the reported microbiome profiles from early infancy [9], [19], [20], [41]. Our data showed that, although at the phylum level, the meconium is more similar to that of infants than to adults, at the genus level, the dominant taxa found in the early infant stool from vaginal deliveries (*Lactobacillus*, *Prevotella*) or from C-sections (*Acinetobacter*, *Propionibacterineae*, *Micrococcineae*, *Corynebacterineae*) [9], [11], [19], [41] were not prevalent (<1%) in over 90% of the meconium samples.

### Diversity of the Meconium Microbiota

The average microbiota diversity within the meconium microbiome was lower than that of adult feces (3.1 vs. 8.5, *p* = 1.7e-4, **Figure 1C**). Within meconium samples, the DM group showed higher alpha-diversity (within-sample bacterial diversity) than that from the no-diabetes or GDM groups (6.2 vs. 3.1 and 2.9; FDR-adjusted *p* = 1e-3 and 0.08, respectively). Meconium samples of babies born to mothers with no diabetes or GDM had a higher variability in beta-diversity (between-sample bacterial diversity) than the DM group (*p* = 0.006 and 0.0017 by F-test, respectively) or the adult samples (*p* = 3e-5 and 2.3e-4 by F- test, respectively) (**Figure S2A in File S1**). The DM group showed a lower variability than the adult samples.

### Meconium Microbiome and Maternal Diabetes Status

**Figure 3:**
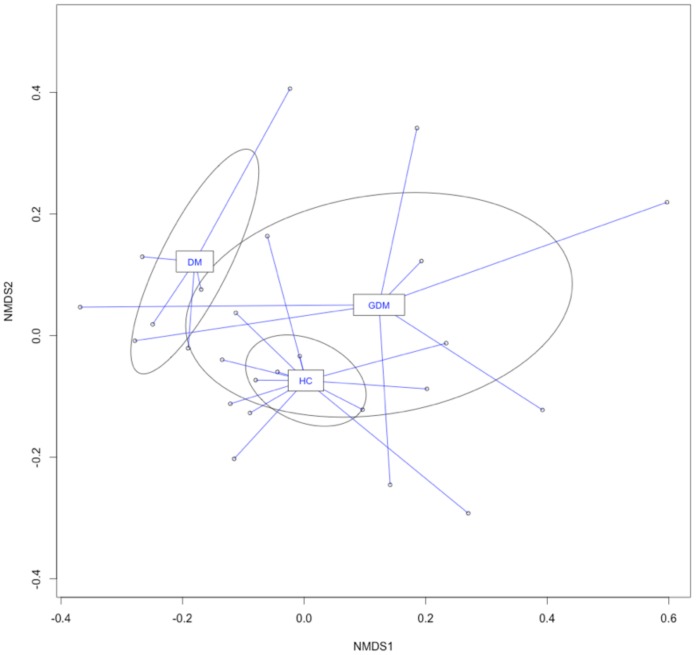
Comparison of meconium microbiota from neonates born to HC (healthy), DM (type 2 diabetes) and GDM (gestational diabetes) mothers using nMDS ordination. The unweighted UniFrac distance matrices generated from QIIME were visualized in nMDS plot. Lines connect individual communities to the centroid values for each group. The ellipses were drawn to represent the class standard errors.

**Figure 4:**
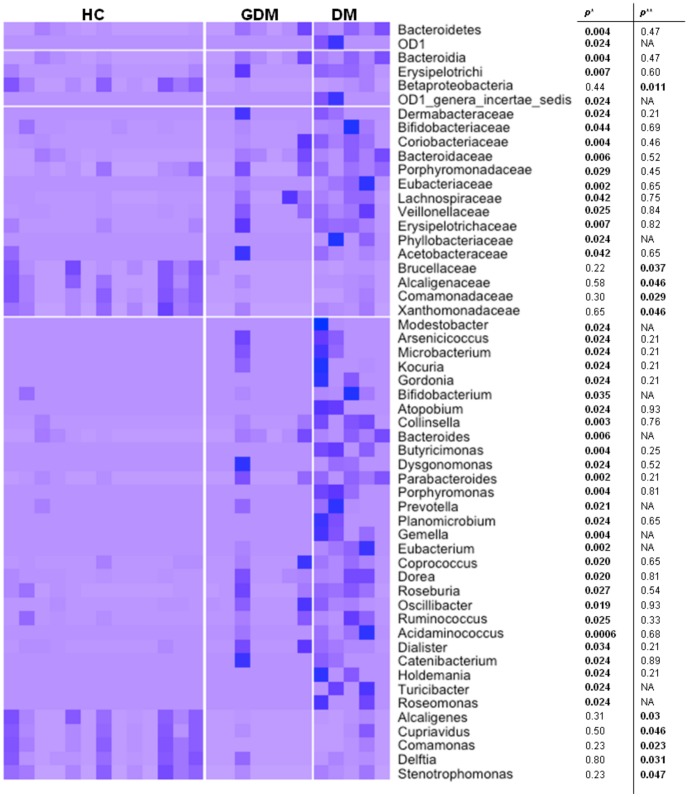
List of OTUs that showed significant difference between neonates from mothers with different diabetes states. HC(healthy) group was combined with both healthy and subclinical groups. *unadjusted p-value from WMW-test between HC and DM(type 2 diabetes) group; **unadjusted p-value from WMW-test between HC and GDM(gestational diabetes) group.

The nMDS analysis using unweighted UniFrac distance matrices showed a considerable separation of the overall microbiota by maternal diabetes status (**Figure 3**). The PerMANOVA test detected that the DM and GDM groups were each significantly different from the no-diabetes group (*p* = 0.004, 0.022, respectively); however no significant difference was observed between the DM and GDM groups (*p* = 0.16). Consistent with the PerMANOVA results, weighted and unweighted UniFrac PCoA showed a separation of the DM from no-diabetes samples on the PC1 *vs.* PC2 plot (**Figure S2B in File S1**), mostly driven by the enrichment of *Bacteroidetes* and reduction of *Proteobacteria* in the DM meconium samples (**Figure S3 in File S1**). Notably, the 4 subjects with subclinical DM (subjects BM3, BM180, BM52 and BM276) showed no separation from other no-diabetes samples (**Figure S3 in File S1**). Furthermore, we compared the abundance of the OTUs among these three groups. **Figure 4** depicts all OTUs that showed significant difference (p<0.05) at the phylum, class, family and genus level between DM and no-diabetes controls. Specifically, several OTUs, such as *Bacteroidetes* (phyla), *Lachnospiraceae* (family) and *Parabacteriodes* (genus) that were enriched in adult Type 2 diabetes patients [42], were also significantly enriched in the meconium samples of babies born to DM mothers compared to the no-diabetes group. No significance was achieved when *p*-values were adjusted for multiple hypothesis testing.

### Relationship between the Meconium Microbiome and Other Maternal and Newborn Characteristics

Previous work has shown the association of the infant microbiome with the delivery mode [9], [20]. In our study, the PerMANOVA test showed no significant difference between the two delivery methods in neonates born to no-diabetes mothers (*p* = 0.26). The weighted and unweighted PCoA plot also showed no separation (**Figure S2C in File S1**). Moreover, we found no significant differences in taxa between the two delivery modes at each OTU level. Despite the small sample size, we further investigated the impact of the type of C-section (i.e., emergency or elective) on newborn's gut microbiota and found no significant differences between the microbiome profiles of the 8 emergency and 2 elective C-sections.

In the univariate regression analysis, we found that the maternal diabetes status was significantly associated with the abundance of *Bacteroidetes* (*p* = 0.029), PC2 (*p* = 0.015) and the overall microbiome profile (*p* = 0.038) (**Table S2 in File S1**). No other demographic or clinical characteristics were found to be associated with the meconium microbiome profile.

## Discussion

We implemented 16S rRNA sequencing using the Pacbio RS system to explore the meconium microbiome of 23 newborns. Our findings showed that the meconium was not sterile in all of our study subjects and contained diverse microbiota communities, regardless of the demographic and clinical characteristics. A number of studies using culture-dependent or independent methods have established the presence of live bacteria in the fetus prior to birth [12], [21]–[26], [30]–[32], [43]. However, until the development of the next generation sequencing (NGS) technology, the detailed characterization of the microbiome at the taxa level was challenging. Moreover, while a number of studies have compared the bacterial composition of the adult stool with early infant stool [9], [11], [19], [20], [28], only a handful of small studies have been carried out in meconium [24], [25], [32]. Using a culture-independent NGS technology, we demonstrated that the meconium microbiota substantially differ from those in adult feces, with the overall microbiome diversity being significantly lower than that of the adult stool (**Figure 1**). While at the phylum level the meconium microbiome was more similar to that of infants [9], [11], [19], [41] than adults, at the genus level, the dominant taxa found in the early infant stool were not prevalent in the meconium samples.

Among the meconium samples, we observed a significant increase in the diversity of various bacterial types if mothers had pre-gestational DM compared to those with no diabetes or GDM.

These results are also consistent with the recent findings that have suggested that certain microbiota components are more prevalent in adult individuals with DM [42], [44]. Specifically, at the OTU level, the *Bacteroides*, *Parabacteroides* and *Lachnospiraceae*, which have been reportedly prevalent in adult diabetes patients [29], [42], were enriched in the meconium of babies born to mothers with DM. Despite the enrichment of certain bacteria, a previous study have reported no difference in the mean OTU diversity within each sample between the adult DM cases and controls [45]. However, in our study, we observed that the meconium in the DM group showed higher alpha-diversity than that in the no-diabetes or GDM groups with a possible explanation being that the bacterial transmission to fetus may be more permissible under maternal diabetic conditions.

An additional illustration of the link between the meconium microbiota composition and maternal and infant health has been provided by Gosalbes *et al*., who reported that the meconium microbiota types dominated by lactic acid or enteric bacteria are differentially associated with maternal eczema and respiratory problems in infants [24]. Moreover, they have shown that some of the species that reach the fetal gastrointestinal tract prenatally can be present in the infant long into the first year, underlining the potential long-term clinical consequences of the initial microbiota content. We speculate that the bacterial diversity in the gut of a fetus may reflect pathophysiological processes occurring during pregnancy or represent a direct transmission of maternal intestinal bacteria. Prospective studies will be required to further clarify the path of the mother-to-baby efflux of commensal microbes during pregnancy, as well as the impact of the maternal microbiome on predisposition to adult-onset diseases.

Microbiome studies of early infancy have demonstrated a significant effect of the mode of delivery on the microbiome composition, suggesting the likely association of the infant gut bacteria with maternal vaginal or skin microbiome habitats [9], [20]. However, in our study, no significant differences in the overall microbiome composition by the mode of delivery were detected in babies born to mothers with and without diabetes. Furthermore, we observed that neither of the dominant OTUs reported previously [9] at the genus level in the stool of young infants delivered vaginally (*Lactobacillus*, *Prevotella*) or via C-section (*Acinetobacter*, *Propionibacterineae*, *Micrococcineae*, *Corynebacterineae*) were prevalent in the majority of the meconium samples. Instead, the most common OTUs at the genus level in the meconium included *Comamonas*, *Escherichia/Shigella*, *Klebsiella* and *Proteus*, which are predominantly aerobic or facultative anaerobic organisms. *Comamonas* was found in human appendix, a small pouch attached to the first portion of the large intestine [46] and *Escherichia/Shigella*, *Klebsiella* and *Proteus* occur naturally in the human gut [47], further supporting our hypothesis that the initial colonization in the human gut starts prior to birth. While our findings are consistent with a recent report that also showed no substantial overlap of the meconium microbiota with maternal habitats, especially in babies born via C-section [24], it contrasts previous analyses demonstrating that the microbiota recovered from multiple newborn sites closely resembles vaginal or skin microbiota, depending on the mode of delivery [9]. Emerging studies point toward pregnancy as the beginning of bacterial exposure for the developing fetus [23]–[25]. A recent study on the microbiome dynamics in the gut of pregnant women has shown that the bacterial composition evolves between the 1^st^ and 3^rd^ trimesters, with the maternal gut microbiome in the 3^rd^ trimester being more similar to what we observed in the meconium, including the increased variability in beta-diversity, the compositional dissimilarity among samples, increasing abundance of *Proteobacteria*, and the reduction in alpha-diversity between samples [48]. Taken together, it further suggests that the maternal microbiota can be transferred to the fetus during pregnancy.

For this study, we used the Pacbio RS system. Compared with other NGS platforms, Pacbio RS has the advantage of a much shorter sequencing time (1 to 2 hours) and the much longer read length (up to 8,000 bases). However, the embedded relatively high random sequencing error rate was until recently considered a limiting factor for applying this technology to 16S-based taxonomy assignment and phylogenetic analysis. Our result showed that using CCS reads we were able to replicate an earlier experiment and correctly assign the OTUs to at least the genus level.

This study's limitations include the fact that the sample size may not have allowed us to detect modest differences in bacterial distribution. Nevertheless, it was comparable to the size of a recent study with similar objectives and design [24] and allowed us to detect significant differences with regard to maternal diabetes status. Also, we did not have access to the matched maternal samples; thus, evidence connecting the fetal microbiome directly to the maternal microbiome is lacking. Future studies comparing bacterial composition of the matched maternal (vaginal, placental and fecal) and neonatal microbiome are warranted to determine the major source of the newborn microbiota. In addition, since the gut microbiota changes dramatically during pregnancy [48], a direct comparison between pregnant and non-pregnant women with DM will be important to explore the effect of pregnancy on bacterial content in the context of DM. Furthermore, numerous prenatal factors, such as chorioamnonitis or premature rupture of membranes, maternal prenatal smoking, maternal stress, and others , may contribute to the initial colonization of the gut microbiome. Women were also excluded from the study if they received antibiotic treatment over the course of pregnancy, precluding testing the relationship between maternal infection, diabetes, and meconium content. However, women undergoing C-section and receiving a prophylactic dose of an antibiotic as a standard of care were retained in the study, which could potentially affect the meconium microbiota, especially if antibiotics were given before cord clamping. The sample size of this study would not allow to systematically assess the effect of these and other factors on the meconium microbiome profile. Larger studies collecting extensive prenatal and neonatal data are underway to explore the role of other factors that have effects on the meconium microbiome. Also, health-related information, such as BMI, maternal smoking or glucose level, was not accessible on several enrolled subjects due to the lack of consent or ethical issues associated with administering GCT to individuals with established DM. Therefore, we were unable to establish the relationship between these traits and the meconium microbial composition. Moreover, the time of sampling widely varied among the newborns raising the possibility that environmental exposures may have influenced the bacterial content for those who passed their first stool at a later time. However, the time of sampling was not significantly different between the study groups; therefore, it is unlikely to systematically bias our results. Also, it is known that maternal diabetes can increase the risk of pre-term birth, due to an increase in both fetal and maternal indications for delivery. In our study, we did not see microbiota differences by gestational age among DM, GDM, and no-diabetes groups; however, future studies with a larger sample size will have to examine whether gestational age at delivery is associated with the meconium microbiota. In addition, we did not account for susceptibility loci predisposing to DM, which could also be transmitted from a mother to an infant and potentially affect the microbiome. Future studies will have to determine if host DM risk alleles are associated with a “diabetic” microbial profile.

In summary, our study provides further evidence that meconium contains diversified microbiota and suggests that the initial colonization of the gut flora may start prior to birth. Furthermore, the meconium microbiome of babies born to DM mothers is enriched for the bacterial OTUs observed in the fecal microbiome of adult DM patients. These findings can enhance our understanding of a non-genetic risk of transmission of DM, and help design novel preventive measures for adult onset diseases.

## Supporting Information

File S1 **File includes Tables S1 and S2, and Figures S1–S3.** Table S1. Sequences of bar-coded primers for PCR to generate 16S sequencing amplicons. Table S2. Association between the microbiome composition and clinical data. *P*-values are shown. Figure S1. Description statistic of Pacbio RS 16S sequencing results. A. Histogram of the counts of reads and CCS (circular consensus sequencing) reads at different read length in three chips. This figure demonstrates that a single run of Pacbio RS for 2×45 minutes generated ∼55 k reads, of which ∼33 k reads were longer than 2 kb. B. Histogram to demonstrate the mean read length of CCS reads is 443 bp (range 400–500). C. Bar plots to compare the taxa classification and abundance of 3 stool samples and their repeated measurements at all 5 taxonomy levels. D. Bar plots to show the misclassification rate of *E.coli* strain at all 5 taxonomy levels. Figure S2. Beta diversity of the microbiome from neonate meconium samples. A. Variability in beta-diversity of the microbiome using unweighted or weighted Unifrac distances. B. Unifrac unweighted and weighted PCoA PC1 *vs* PC2 plot to show the overall similarity of the microbiome of neonates born to mothers with three different diabetic statuses. C. Unifrac unweighted and weighted PCoA plot to show the overall similarity of the microbiome from born via different delivery methods. Figure S3. Biplot of the principal coordinates analysis (PCoA) for stool bacteria comparing the healthy adult (Adult stool, red), neonates born to healthy mothers (HC, green), neonates born to mothers with gestational diabetes(GDM, yellow) and neonates born to mothers with type 2 diabetes(DM, blue). Significant separation can be observed between the DM and HC groups.
(PDF)
