# Supplementary material for: Diversified Microbiota of Meconium Is Affected by Maternal Diabetes Status
Source: PLoS One. 2013 Nov 6;8(11):e78257. doi: 10.1371/journal.pone.0078257 (PMC3819383; doi:10.1371/journal.pone.0078257)

Supplementary Data

Supplementary Table S1. Sequences of bar-coded primers for PCR to generate 16S sequencing amplicons. The barcodes are in lowercase.

Primer Name	Sequence
347F	GGAGGCAGCAGTRRGGAAT
803R_MID1	gcgctctgtgtgcagcCTACCRGGGTATCTAATCC
803R_MID2	tcatgagtcgacactaCTACCRGGGTATCTAATCC
803R_MID3	tatctatcgtatacgcCTACCRGGGTATCTAATCC
803R_MID4	atcacactgcatctgaCTACCRGGGTATCTAATCC
803R_MID5	acgtacgctcgtcataCTACCRGGGTATCTAATCC
803R_MID6	tgtgagtcagtacgcgCTACCRGGGTATCTAATCC
803R_MID7	agagacacgatactcaCTACCRGGGTATCTAATCC
803R_MID8	ctgctagagtctacagCTACCRGGGTATCTAATCC
803R_MID9	agcactcgcgtcagtgCTACCRGGGTATCTAATCC
803R_MID10	tcatgcacgtctcgctCTACCRGGGTATCTAATCC
803R_MID11	agagcatctctgtactCTACCRGGGTATCTAATCC
803R_MID12	cgcatcgactacgctaCTACCRGGGTATCTAATCC
803R_MID13	cgtagcgtgctatcacCTACCRGGGTATCTAATCC
803R_MID14	atgctgatgactgcgaCTACCRGGGTATCTAATCC
803R_MID15	tgcgtgagctgtacatCTACCRGGGTATCTAATCC
803R_MID16	cgatcatctatagacaCTACCRGGGTATCTAATCC

Supplementary Table S2. Association between the microbiome composition and clinical data. *P*-values are shown.

Clinical Variables/Microbiome composition	Unweighted principle components			Representative taxa			unweighted unifrac distance matrices*
	PC1	PC2	PC3	Bacteroidetes	Firmicutes	Proteobacteria	
Baby Sex	0.40	0.36	0.24	0.31	0.91	0.89	0.34
TOS[1](hrs)	0.29	0.84	0.67	0.52	0.95	0.92	0.29
Delivery Status	0.07	0.19	0.07	0.26	0.85	0.95	0.07
BMI1[2]	0.98	0.52	0.06	0.28	0.66	0.62	0.54
Gestational age	0.60	0.46	0.49	0.43	0.63	0.61	0.98
Disease Status	0.09	***0.015***	0.65	***0.029***	0.70	0.41	***0.038***

***** Multivariate analysis was performed using function [envfit] in ***vegan*** package in *R* to fit clinical variables onto an ordination based on UniFrac distance matrices;

[1] TOS=Time of sample collection;

[2] BMI1=body mass index(BMI) in 1st trimester.

Figure S1. Description statistic of Pacbio RS 16S sequencing results. **A**. Histogram of the counts of reads and CCS (circular consensus sequencing) reads at different read length in three chips. This figure demonstrates that a single run of Pacbio RS for 2x45 minutes generated ~55k reads, of which ~33k reads were longer than 2kb. **B**. Histogram to demonstrate the mean read length of CCS reads is 443bp (range 400-500). **C**. Barplots to compare the taxa classification and abundance of 3 stool samples and their repeated measurements at all 5 taxonomy levels. **D**. Barplots to show the misclassification rate of *E.coli* strain at all 5 taxonomy levels.

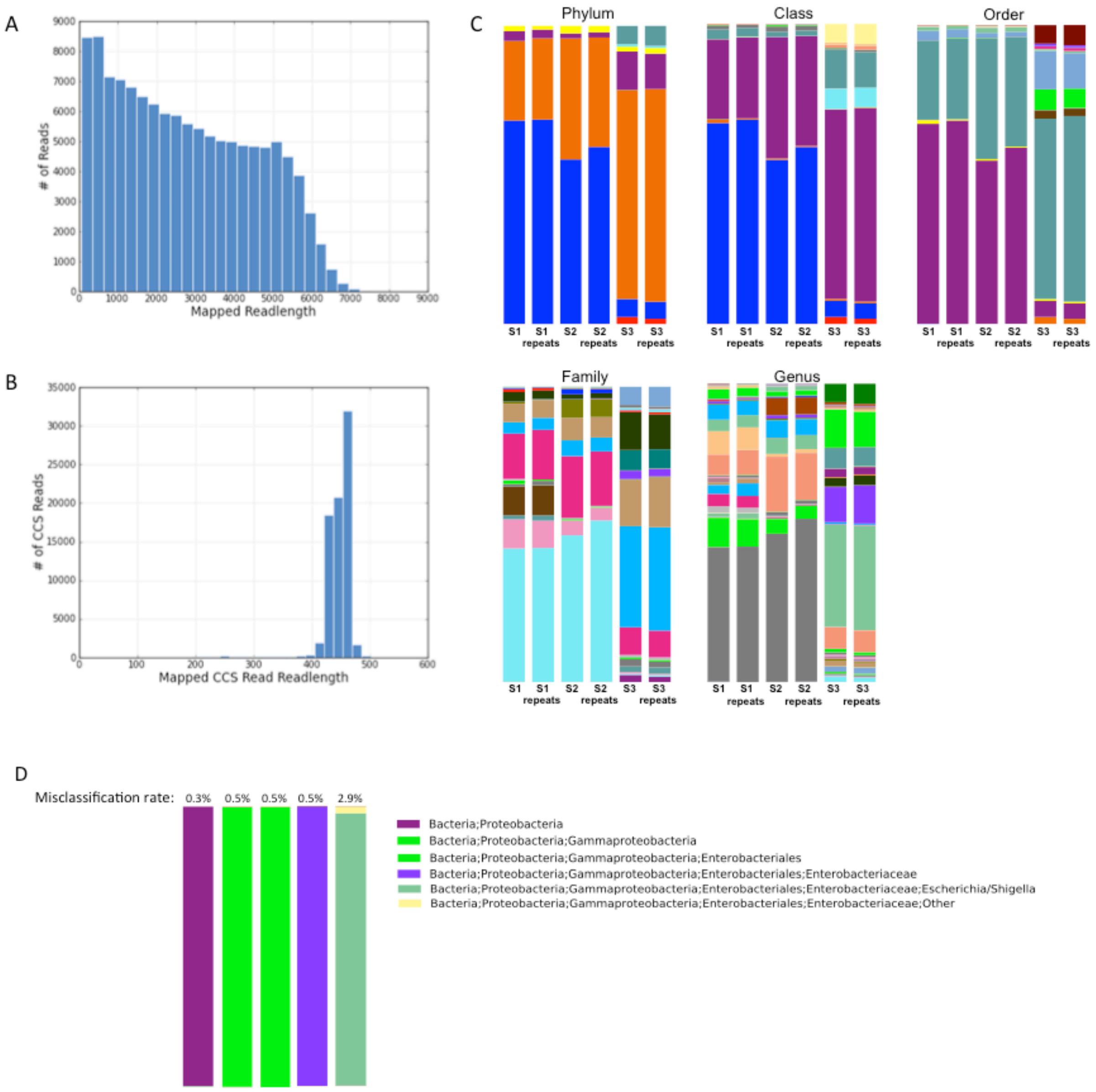

Figure S2. Beta diversity of the microbiome from neonate meconium samples. A. Variability in beta-diversity of the microbiome using unweighted or weighted Unifrac distances. **B**. Unifrac unweighted and weighted PCoA PC1 *vs* PC2 plot to show the overall similarity of the microbiome of neonates born to mothers with three different diabetic statuses. **C**. unifrac unweighted and weighted PCoA plot to show the overall similarity of the microbiome from born via different delivery methods.

A

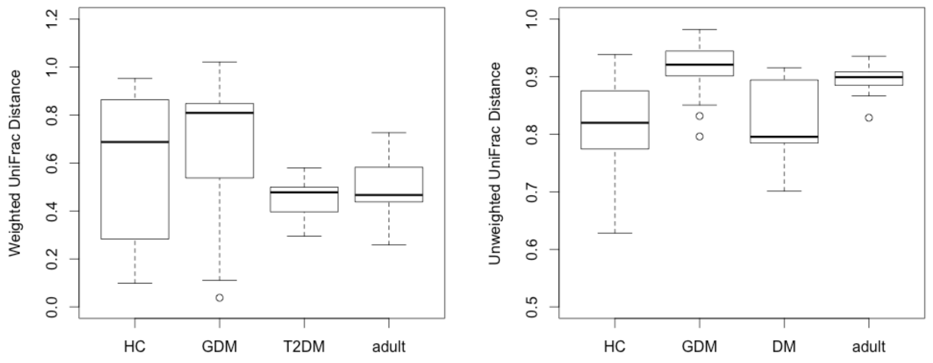

B

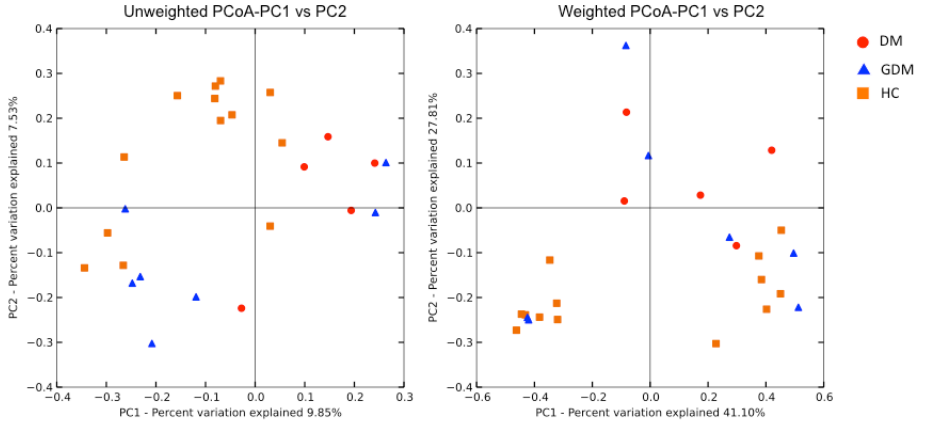

C

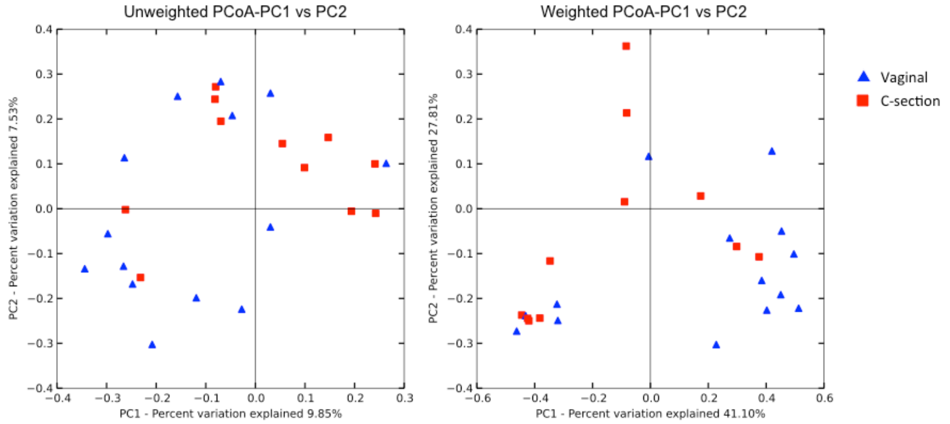

Figure S3. Biplot of the principal coordinates analysis (PCoA) for stool bacteria comparing the healthy adult (Adult stool, red), neonates born to healthy mothers (HC, green), neonates born to mothers with gestational diabetes(GDM, yellow) and neonates born to mothers with type 2 diabetes(DM, blue). Significant separation can be observed between the DM and HC groups.

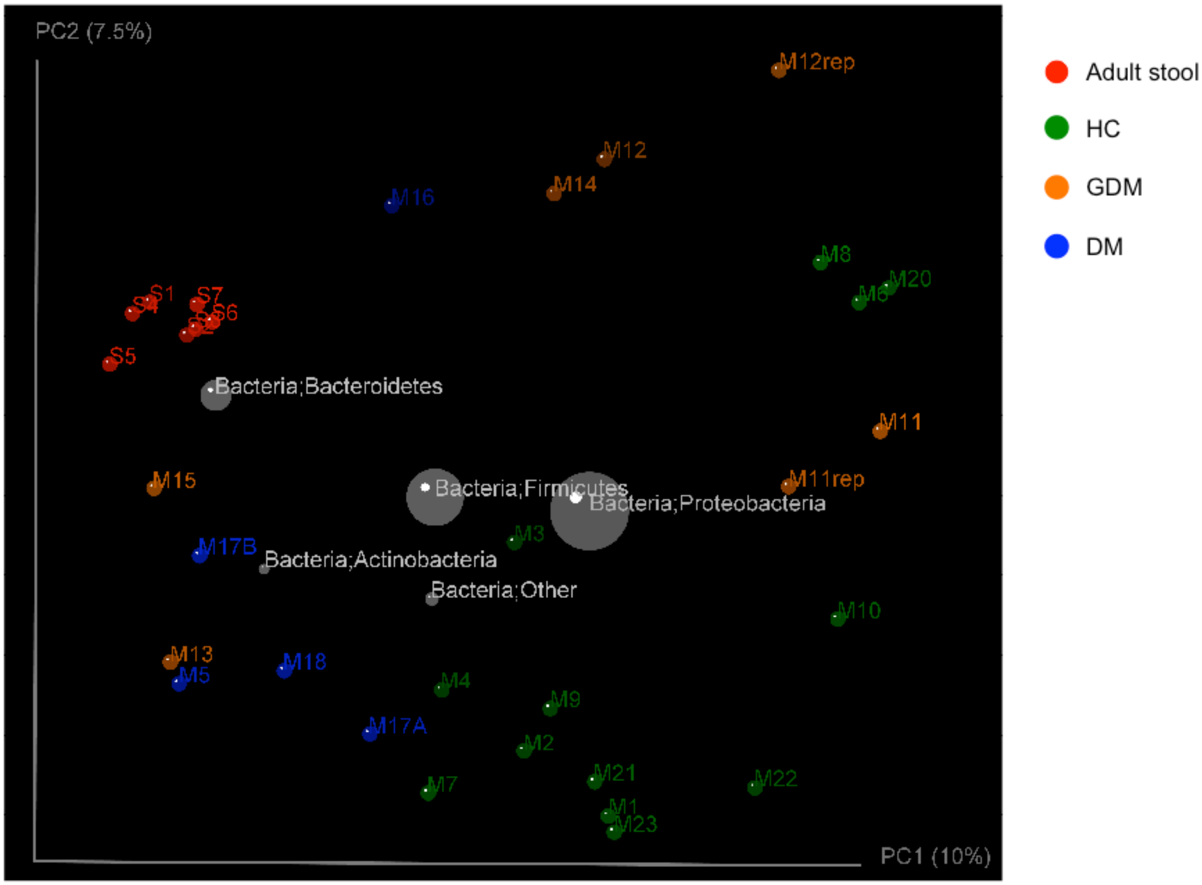

Supplement: File S1 — File includes Tables S1 and S2, and Figures S1–S3. Table S1. Sequences of bar-coded primers for PCR to generate 16S sequencing amplicons. Table S2. Association between the microbiome composition and clinical data. P-values are shown. Figure S1. Description statistic of Pacbio RS 16S sequencing results. A. Histogram of the counts of reads and CCS (circular consensus sequencing) reads at different read length in three chips. This figure demonstrates that a single run of Pacbio RS for 2×45 minutes generated ∼55 k reads, of which ∼33 k reads were longer than 2 kb. B. Histogram to demonstrate the mean read length of CCS reads is 443 bp (range 400–500). C. Bar plots to compare the taxa classification and abundance of 3 stool samples and their repeated measurements at all 5 taxonomy levels. D. Bar plots to show the misclassification rate of E.coli strain at all 5 taxonomy levels. Figure S2. Beta diversity of the microbiome from neonate meconium samples. A. Variability in beta-diversity of the microbiome using unweighted or weighted Unifrac distances. B. Unifrac unweighted and weighted PCoA PC1 vs PC2 plot to show the overall similarity of the microbiome of neonates born to mothers with three different diabetic statuses. C. Unifrac unweighted and weighted PCoA plot to show the overall similarity of the microbiome from born via different delivery methods. Figure S3. Biplot of the principal coordinates analysis (PCoA) for stool bacteria comparing the healthy adult (Adult stool, red), neonates born to healthy mothers (HC, green), neonates born to mothers with gestational diabetes(GDM, yellow) and neonates born to mothers with type 2 diabetes(DM, blue). Significant separation can be observed between the DM and HC groups. (PDF) [file pone.0078257.s001.pdf]
